# Structure-Activity Relationships of Constrained Phenylethylamine Ligands for the Serotonin 5-HT_2_ Receptors

**DOI:** 10.1371/journal.pone.0078515

**Published:** 2013-11-07

**Authors:** Vignir Isberg, James Paine, Sebastian Leth-Petersen, Jesper L. Kristensen, David E. Gloriam

**Affiliations:** Department of Drug Design and Pharmacology, Faculty of Health and Medical Sciences, University of Copenhagen, Copenhagen, Denmark; University of Edinburgh, United Kingdom

## Abstract

Serotonergic ligands have proven effective drugs in the treatment of migraine, pain, obesity, and a wide range of psychiatric and neurological disorders. There is a clinical need for more highly 5-HT_2_ receptor subtype-selective ligands and the most attention has been given to the phenethylamine class. Conformationally constrained phenethylamine analogs have demonstrated that for optimal activity the free lone pair electrons of the 2-oxygen must be oriented syn and the 5-oxygen lone pairs anti relative to the ethylamine moiety. Also the ethyl linker has been constrained providing information about the bioactive conformation of the amine functionality. However, combined 1,2-constriction by cyclization has only been tested with one compound. Here, we present three new 1,2-cyclized phenylethylamines, **9**–**11**, and describe their synthetic routes. Ligand docking in the 5-HT_2B_ crystal structure showed that the 1,2-heterocyclized compounds can be accommodated in the binding site. Conformational analysis showed that **11** can only bind in a higher-energy conformation, which would explain its absent or low affinity. The amine and 2-oxygen interactions with D3.32 and S3.36, respectively, can form but shift the placement of the core scaffold. The constraints in **9**–**11** resulted in docking poses with the 4-bromine in closer vicinity to 5.46, which is polar only in the human 5-HT_2A_ subtype, for which **9**–**11** have the lowest affinity. The new ligands, conformational analysis and docking expand the structure-activity relationships of constrained phenethylamines and contributes towards the development of 5-HT_2_ receptor subtype-selective ligands.

## Introduction

The neurotransmitter serotonin (5-hydroxytryptamine, 5-HT) has key roles in mood, libido, aggression, anxiety, cognition, sleep, appetite and pain and also regulates peripheral functions in the cardiovascular, gastrointestinal, endocrine and pulmonary system.[1]-[4] Serotonergic ligands have proven effective drugs in the treatment of migraine, pain, obesity, and a wide range of psychiatric and neurological disorders.[1], [5]–[9] The serotonergic system comprises 12 Class A G protein-coupled receptors and one ligand-gated ion channel that together are divided into 7 pharmacological subfamilies. The 5-HT_2_ subfamily consists of the three subtypes, serotonin receptors 2_A-C_ (5-HT_2A-C_). 5-HT_2A_ inhibition by clinical drugs has antipsychotic (e.g., clozapine) and antidepressive (e.g., mianserin) effects.[10] 5-HT_2A_ subtype stimulation by full or partial agonists mediates the hallucinogenic effects of many natural (e.g. psilocybin and mescaline) and synthetic drugs.[1], [11], [12]

**Figure 1:**
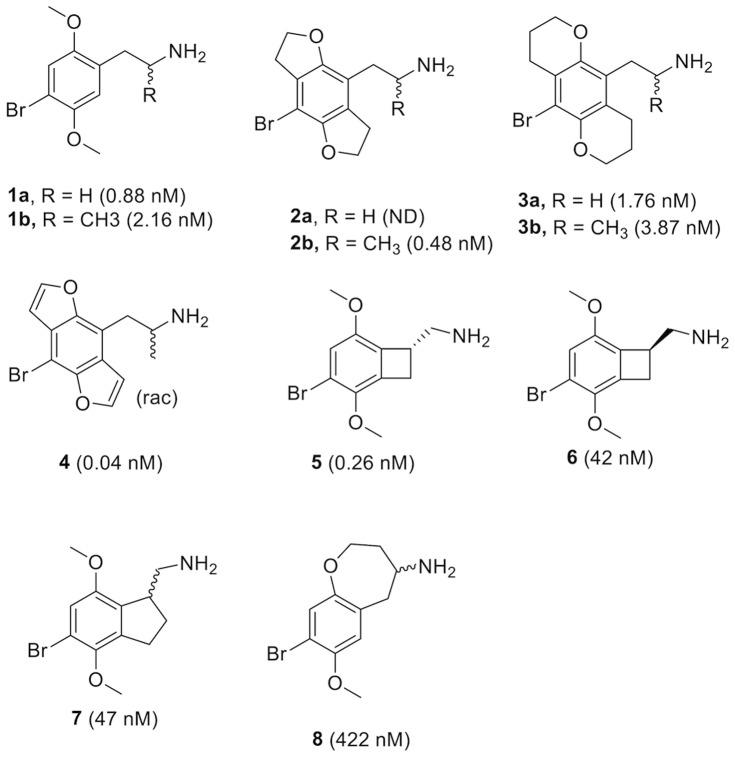
Published conformationally restrained analogs of 1. 5-HT_2A_ affinities are given within parenthesis.

The 5-HT_2A_ agonist structures generally fall into one of three categories, phenethylamines, tryptamines and ergolines.[13] There is a clinical need for more highly 5-HT_2_ subtype-selective ligands and the most attention has been given to the phenethylamine class. The phenethylamine ligand 2C-B (**1a** in Fig. 1) contains the structural features required for hallucinogenic activity; a primary amine separated from the phenyl ring by two carbon atoms, 2- and 5-aromatic methoxy groups, and a hydrophobic 4-substituent. Methylation of the amine α-carbon, as in DOB (**1b**), DOB-fly (**2b**) and DOB-butterfly (**3b**), results in slightly decreased in vitro affinities but increases the strength and duration of the response in vivo – hypothesized to be a consequence of increased metabolic stability resulting in higher exposure.[14]

**Figure 2:**
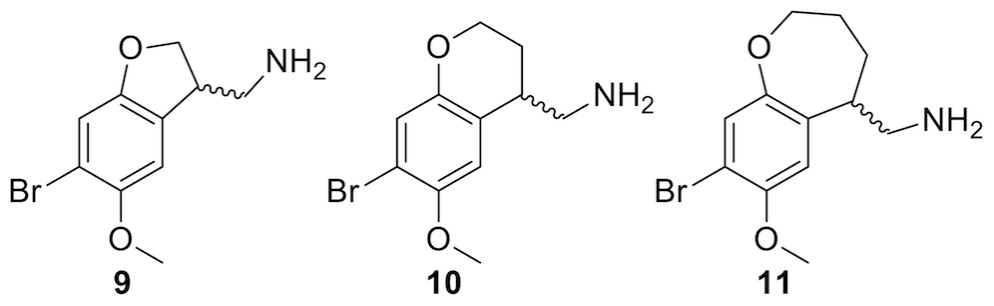
The new conformationally constricted analogs reported in this study.

Conformationally constrained analogs, primarily **2**–**4**, have demonstrated that for optimal activity the free lone pair electrons of the 2-oxygen must be oriented syn and the 5-oxygen lone pairs anti relative to the ethylamine moiety.[15]–[17] Mutagenesis and ligand structure-activity data suggest that the 2- and 5-oxygen atoms hydrogen bond to serine residues, S3.36^159^ and S5.43^239^, respectively.[18], [19] Also the ethyl linker has been constrained, exemplified by **5**–**7**, providing information about the bioactive conformation of the amine functionality.[20] Combined 1,2-constraint by cyclization has only been tested with one compound, **8**, which exhibits 373-fold lower affinity than the unconstrained reference DOB (**1a**).[21] Here, we set out to further explore the structure-activity relationships of 1,2-cyclized phenethylamine ligands. The analysis includes the synthesis of three new compounds, **9**–**11** (Fig. 2), binding affinity measurements, conformational analysis, receptor homology modeling and ligand docking.

## Results

### Synthes of the 1,2-cyclized phenethylamines 9–11

**Figure 3:**
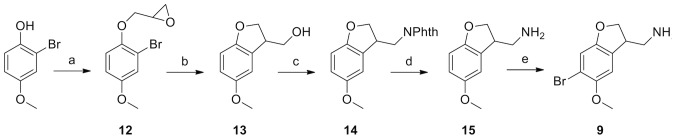
Synthesis of 9. Reagents and Conditions: (a) epichlorohydrin, Cs_2_CO_3_, MeCN, reflux, 4 h; (b) BuLi, THF, −78°C to r.t., 30 min; (c) phthalimide, PPh_3_, DEAD, CH_2_Cl_2_, r.t., 1 h; (d) N_2_H_4_.H_2_O, EtOH, reflux, 2 h; (e) Br_2_, AcOH, r.t., 18 h.

**Figure 4:**
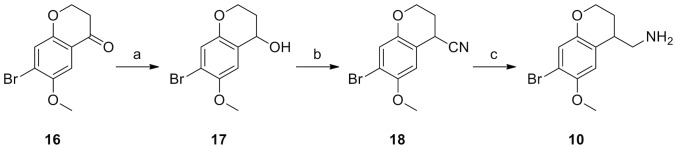
Synthesis of 10. Reagents and Conditions: (a) NaBH_4_, EtOH, r.t., 2 h; (b) Me_3_SiCN, BF_3_.Et_2_O, CH_2_Cl_2_, −78°C to r.t.; (c) DIBALH, THF, reflux, 2 h. The 7-bromochroman-4-one 16 was prepared as previously described.[23]

**Figure 5:**
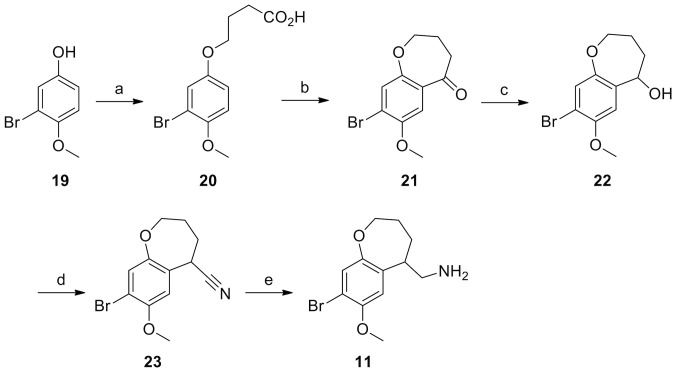
Synthesis of 11. Reagents and Conditions: (a) Ethyl 3-bromobutyrate, Cs_2_CO_3_, MeCN, reflux, 2 hrs; (b) polyphosphoric acid, 90°C for 1 h. (c) NaBH_4_, EtOH, r.t., 2 h; (d) Me_3_SiCN, BF_3_.Et_2_O, CH_2_Cl_2_, −78°C to r.t.; (e) DIBALH, THF, reflux, 2 h.

The synthetic routes of **9**-**11** are shown in Figures 3-5 and described in detail in Methods S1 (Supporting information). Briefly, **9** was prepared starting from commercially available 2-bromo-4-methoxyphenol, epoxide **12** underwent 5-exo cyclisation to dihydrobenzofuranyl methanol **13** upon treatment with BuLi, as reported by Bradsher.[22] Introduction of the amino group in **14** was accomplished by a Mitsunobu reaction with phthalimide. This was followed by deprotection to give the free amine **15** and finally 4-bromination to yield **9**.

Compounds **10** and **11** could not be prepared in the same manner as **9** because the required 6-exo/7-exo cyclisations onto the corresponding epoxides did not occur. We were thus forced to incorporate the bromine at an earlier stage to circumvent this problem. **10** was prepared as shown in Figure 4. The 7-bromochroman-4-one **16** was prepared as previously described[23] and reduced with sodium borohydride to alcohol **17**. Reaction with trimethylsilyl cyanide afforded nitrile **18**, which was reduced with diisobutylaluminiumhydride (DIBALH) to the amine in **10**.

**11** was synthesized as shown in Figure 5. Bromophenol **19** [24] was alkylated using ethyl 3-bromobutyrate and Cs_2_CO_3_ in refluxing acetonitrile. Cyclization of the resulting acid **20** via treatment with polyphosphoric acid afforded dihydrobenzoxepinone **21**, which in turn gave access to amine **11** following the same protocol utilized in the synthesis of **10**: borohydride reduction, cyanation and DIBALH reduction.

### Binding affinities

Table 1 shows the binding affinities of published (**1**−**8**) and new (**9**−**11**) 2-oxygen- and/or amine-constrained phenethylamine ligands. The binding affinities of **9**−**11** against the 5-HT_2A-C_ receptors were determined in competition assays with [^3^H]-ketanserin, [^3^H]-LSD and [^3^H]-mesulergine as radioligands for 5-HT_2A_, 5-HT_2B_ and 5-HT_2C_, respectively. **9** and **10** have higher affinities in 5-HT_2B-C_ than 5-HT_2A_. This was unexpected as the 5-HT_2A_ and 5-HT_2C_ affinities are typically the most similar. The highest affinity, 70 nM, is displayed by **10** in 5-HT_2B_. **11** is inactive in 5-HT_2A_ and 5-HT_2C_ and displays only weak affinity (1.9 µM) for 5-HT_2B_.

**Table 1:** Binding affinities of published (1−8) and new (9−11) compounds at human 5-HT_2_ receptors.

		Affinity, K_i_ (nM)				
Compound		5-HT_2A_	5-HT_2B_	5-HT_2C_	Species	Ref.
1a	2C-B	0.88	NA	NA	Human	[20]
1b	DOB	2.16	NA	2.82	Rat	[31]
2a	2C-B-fly	NA	NA	NA	Human	[15]
2b	DOB-fly	0.48	1.60	0.30	Human	[15]
3a	2C-B-butterfly	1.76	NA	1.52	Rat	[16]
3b	DOB-butterfly	3.87	NA	1.85	Rat	[16]
4	Bromo-DragonFLY	0.04	0.19	0.02	Human	[17]
5	TCB-2	0.26	NA	NA	Human	[20]
6		42	NA	NA	Human	[20]
7		47	NA	NA	Human	[20]
8		422	NA	NA	Rat	[21]
9		1040±188	196±28	135±31	Human	
10		847±79	70±13	124±9	Human	
11		>10000	1872±345	>10000	Human	

NA: Not Available

**8**, despite the 7-membered ring, appears to have somewhat higher affinity (422 nM) than **9**−**11**. Of note however, **8** was tested in rat 5-HT_2A_, in which the binding site bears more resemblance to that of 5-HT_2B-C_ as these three receptors contain an alanine in position 5.46 whereas human 5-HT_2A_ holds a more polar serine residue. Thus, until **8** has been tested in human 5-HT_2A_ or 5-HT_2B-C_ we consider it equipotent to **9**−**10**. Also ligands **1b** and **3a**-**b** have been tested in rat receptors and may not be equipotent if tested in human receptors.

### Structure-Activity Relationships

The 1,2-heterocyclized analogs **8**−**11** display at best a 480-fold lower affinity at 5-HT_2A_ than the unconstrained reference 2C-B (**1a**). However, pharmacological testing of **9**−**11** against all three 5-HT_2_ subtypes, revealed significantly higher affinities for the 5-HT_2B_ and 5-HT_2C_ receptors. Below we set out to rationalize these two findings by ligand conformational analysis and, for the first time, ligand docking inside the 5-HT_2B_ crystal structure.[25] Specifically, the different sections have investigated ligand-receptor interactions, ligand conformational penalties of binding and the optimal positions of the 2-oxygen and amine functionalities in comparison to the highest affinity reference compounds, **4** and **5**, respectively.

### The receptor binding site can accommodate 7-membered 1,2-heterocycles

The 5-HT_2B_ receptor has been crystallized in complex with a partial agonist, ergotamine.[25] The reference ligands Bromo-DragonFLY (**4**) and 2C-TCB (**5**) could be docked directly into this crystal structure, but a small optimization of the binding pocket was needed to adapt it to the phenyl-ethylamine scaffold. The contacts for the charged amine, phenyl ring and 4-bromo functionalities were all in perfect alignment with the interaction map of the binding site. The triple-ring core of 2C-TCB (**5**) was better accommodated by tilting F6.52^341^ slightly towards TMH5 to the same position as observed in the G protein-bound β_2_ adrenergic structure.[26] The previously described[19] hydrogen bonding between the 2-oxygen functionality and S3.36^139^ required rotation of the oxygen dihedral towards TMH5 until close (0.6 Å) to the most frequent state (42%) in the library. The proposed hydrogen bond[18] between the 5-oxygen and S5.43^222^ hydroxyl cannot form as the oxygen atom pair distances are 5.8 and 5.7 Å for Bromo-DragonFLY (**4**) and 2C-TCB (**5**), respectively). Inspection of the crystal structure shows that the base (i.e. C-alpha to C-beta bond) of the S5.43^222^ side chain projects towards TMH6 rather than TMH3 and that F6.52^341^ blocks access.

**Figure 6:**
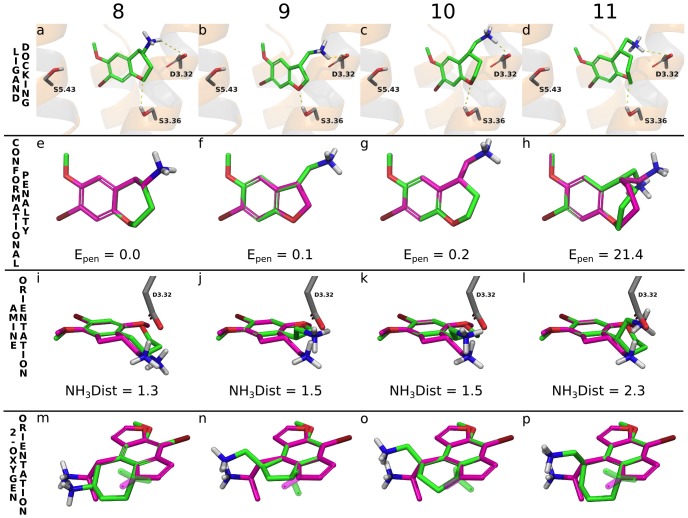
Ligand docking and conformational analyses. **a-d)** Docking poses for **8**−**11** the 5-HT_2B_ crystal structure. **8**−**11** have similar contacts as the reference compounds **4** and **5** for the charged amine, and phenyl ring, whereas the 4-bromo points deeper and closer to A5.46^225^. **e-h)** The docked poses of **8**−**11** (green carbons) overlaid on their calculated lowest energy conformations (magenta carbons). **11** has a high conformational energy penalty, E_pen_ (21.4 kJ/mol) upon binding, which is consistent with its lack of or low affinity for 5-HT_2A-C_. **i-l)** Comparison of the positions of the amine side chains of **8**−**11** (green carbons) superimposed onto the docked reference **5** (magenta carbons). NH_3_Dist is the distance (Å) between the amines of **8**−**11**, respectively, and **5**. The distance is greatest for **11**, indicating a conformationally strained amine side chain upon binding. **m-p)** Comparison of the lone pair orientations of the 2-oxygens of **8**−**11** (green carbons) and **4** (magenta carbons). The lone pair vectors (semi-transparent sticks) of **8**−**11** all differ significantly from **4**. All superimpositioning (Fig. 6e-p) was made on the phenyl, bromine, 2-oxygen and 5-oxygen atoms.

Also the 1,2-cyclized compounds **8**−**11** could be docked directly into the 5-HT_2B_ structure (Figure 6a-d). Similar contacts were achieved for the charged amine and phenyl ring, whereas the 4-bromo pointed deeper and closer to A5.46^225^. Their 2-oxygen lone pairs are directed in opposite direction compared to the reference ligands and the optimal hydrogen bonding angle was found to be for the third rotameric state of S3.36^139^ (21% frequency in rotamer library), which positions the hydroxyl deeper and just below the ligands. For compounds **8** and **9** both enantiomers fitted, although (S)-**8** and (R)-**9** formed more optimal receptor interactions. For **10** and **11** only the R-enantiomer fitted in a way that the 2- and 5-oxygens could be directed towards the corresponding receptor contacts. In conclusion, all compounds could be docked into the 5-HT_2B_ receptor. The 2-oxygen to S3.36^139^ hydrogen bond could form, but required alternative rotamer shifts. A 5-oxygen to S5.43^222^ hydrogen bond could not be formed. Arguably, it may form in another conformational receptor state, but it is unlikely that the helical backbones would move enough. If such as bond is formed it could however be indirect being bridged either by a water molecule or the proximal residue N6.55^344^.

### The inactive compound 11 exhibits a high conformational penalty of binding

We calculated the conformational energy penalties of binding for **8**−**11** by comparing the energies of the receptor-bound poses with their respective lowest energy conformation in solution (Fig. 6e-h). The inactive **11** exhibits a considerable penalty, 21.4 kJ/mol, whereas **8**−**10** display close to none. A closer inspection of **8** and **11**, which both have 7-membered rings shows that their ring conformations are exactly the same in the global energy minimum, whereas their amine positions differ by 2.7 Å. In the docking, **11** displays a higher energy ring conformation, which is necessary to direct the amine in the proximity of D3.32 and also has a strained methyl amine linker. As the binding sites of 5-HT_2A-C_ are identical in the region around this ring, this observation provides a plausible explanation also to the lack of affinity of **11** in the 5-HT_2A_ and 5-HT_2C_ receptors.

### 1,2-cyclization alters the amine and 2-oxygen lone pair orientations and shifts the overall poses

We next investigated the orientation of the amine functionality. Figure 6i-l shows a superimposition of the minimum energy conformations of **8**−**11** to that of docked (R)-TCB-2 (**5**), which is the amine-constrained ligand with the highest affinity (5-HT_2A_: 0.26 nM). In **8** the amine is slightly distanced whereas in **9**−**11** it is positioned closer towards the side of the interacting D3.32. The distances between the charged nitrogen atoms are 1.3, 1.5, 1.5 and 2.3 Å for **8**−**11**, respectively, from that of **5**. After docking, the distances are 0.5−3.2 Å, and the amine is shifted primarily upwards compared to TCB-2 (**5**). We next turned to the lone pair orientations of the 2-oxygen, which has been suggested based on mutagenesis to form a hydrogen bond with S3.36.[19] Figure 6m-p shows a superimposition of the minimum energy conformations of **8**−**11** to that of the docked Bromo-DragonFLY (**4**), which is the 2-oxygen-constrained ligand with the highest affinity (0.02−0.19 nM in 5-HT_2A-C_). The distances between the **5** and **8**−**11** 2-oxygen atoms are small (0.2 Å in **8**,**10**−**11** and 0.5 in **9**). However, as expected from their 2D structures, the orientations of the lone pair vectors differ markedly. This has an effect on the docked poses (Fig. 1a-d), in which the 2-oxygen atoms of **8**−**11** have shifted 1.5−3.0 Å from that of Bromo-DragonFLY (**4**) away from THM5 and a somewhat higher.

The large changes in the amine orientations of **8**−**11** seem to be accommodated by the receptor as the interacting residue D3.32 offers a large contact area and there is some flexibility on both sides (one-carbon linkers in the amine and carboxylic acid). Maintaining the 2-oxygen hydrogen bond to S3.36 seems more challenging, as there is less flexibility at this point. Moreover, the amine and 2-oxygen both interact with residues on the same helix, TMH3, and a helical movement would therefore not relieve the combined constraint. The 1,2-cyclization is therefore compensated for by a translation of the ligand that shifts the positions of the methoxy, bromine and phenyl functionalities and, in particular, the 4-bromo and 5-oxygen substituents are located markedly deeper. Taken together, the constrained moieties may to some extent be compensated for by flexible receptor contact points, but alter the position and/or angle of the core scaffold and so modulate the remote 4-bromine and 5-oxygen functionalities.

### Differences in the binding site may explain the lower affinity of 8−11 in 5-HT_2A_ and higher affinity of 10−11 in 5-HT_2B_

**Figure 7:**
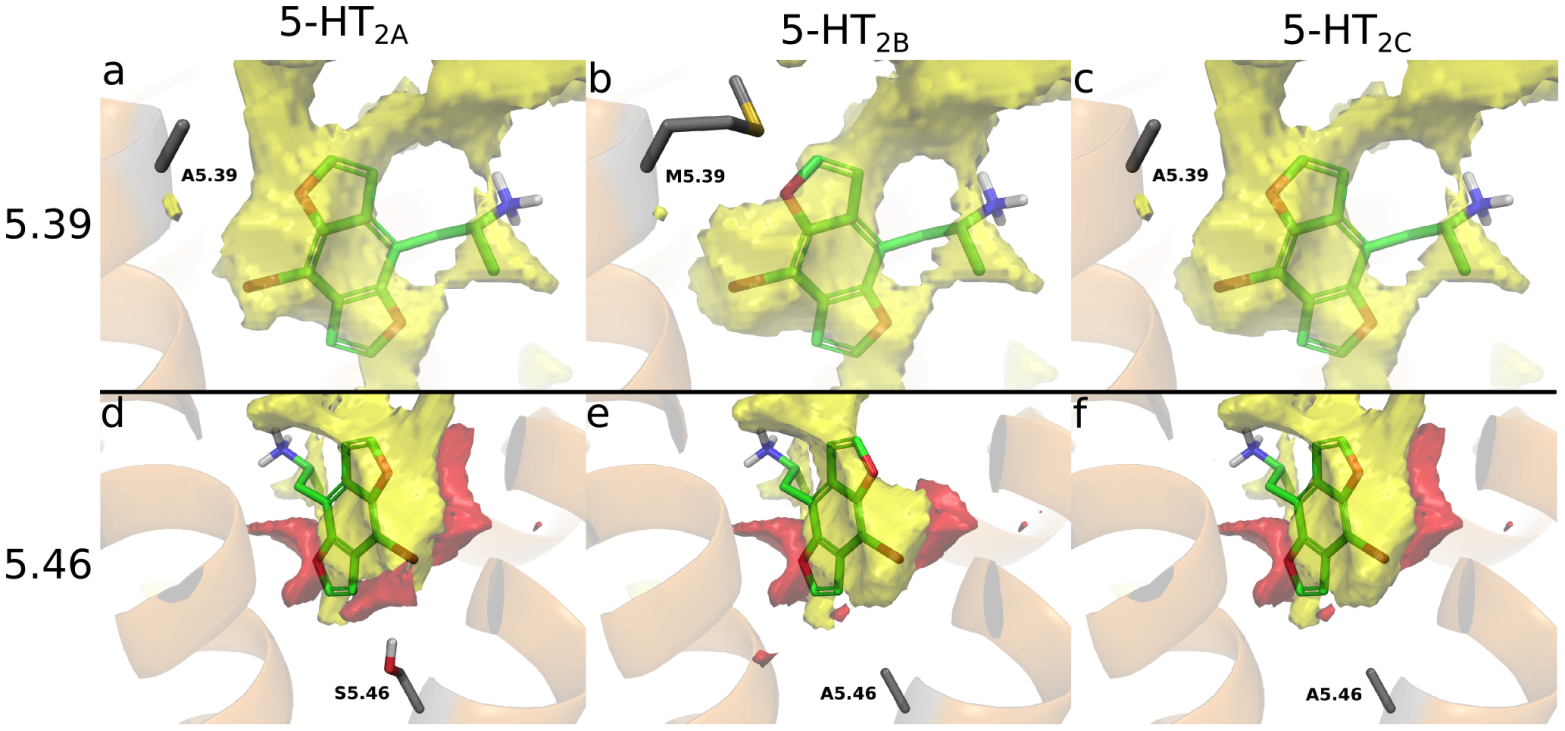
Visualizations of the hydrophobic- (yellow surface) and hydrogen bond acceptor areas (red surface) of the binding sites in 5-HT_2A-C_. The maps were produced with SiteMap.[29] The 5.39 methionine in 5-HT_2B_ (b) reduces the size of hydrophobic pocket. The 2-oxygen matches the hydrogen bond acceptor area for S3.36 interaction whereas the 5-oxygen cannot reach that of S5.43 (left and right sides, respectively, in d-f). 5.46 holds a serine in 5-HT_2A_ (d) resulting in an hydrogen bond acceptor site close to TMH5, where the other receptor subtypes display hydrophobic areas.

**9**−**10** display 5−12 fold higher affinities in 5-HT_2B-C_ than 5-HT_2A_. Arguably, this difference is too large to only be due to the use of different radioligands; an antagonist for 5-HT_2A_ (ketanserin) and agonists for 5-HT_2B_ (LSD) and 5-HT_2C_ (mesulergine). An additional factor is the difference in the binding sites. As noted above human 5-HT_2A_ holds a polar serine residue in 5.46, whereas 5-HT_2B-C_ have an alanine (Fig. 7d-f). In our ligand docking, the 4-bromo substituent is closer to 5.46 in the 1,2-cyclized compounds **8**−**11**. Thus, it is plausible that the lower affinity of these ligands in 5-HT_2A_ is caused by a less favorable environment for the 4-bromine in the presence of S5.46. In future studies it would therefore be interesting to exchange the 4-bromine for a polar substituent, for example a hydroxyl or nitrile, too see if the affinity profile is inverted (i.e. higher affinity in 5-HT_2A_ than 5-HT_2B-C_).

5.39, located approximately two helical turns higher on TMH5, is close to the 5-oxygen of the docked phenethylamine ligands. This position is occupied by a methionine in 5-HT_2B_, but an alanine in 5-HT_2A_ and 5-HT_2C_ (Fig. 7a-c). This could give more room for large ligands in the latter receptors tentatively explaining the somewhat lower affinities of Bromo-DragonFLY (**4)** and DOB-fly (**2b**) in 5-HT_2B_ (Table 1). Taken together, the two subtype differences could explain some of the observed affinity profiles, but many ligands have only been tested in 5-HT_2A_ making it difficult to define any general relationships for all constrained phenethylamines. Future ligand design aiming at selectivity, could exploit the subtype differences.

## Conclusions

Previously the effect of 1,2-cyclization of phenethylamines had only been explored with one ligand (**8**) [21]. Here, we have presented three new 1,2-cyclized of phenethylamines and described their synthetic routes giving access to novel derivatives. The 1,2-heterocyclized analogs **8**−**11** display at best 480-fold lower affinity at 5-HT_2A_ than the unconstrained reference 2C-B (**1a**, Table 1). The ligand docking results show that all four 1,2-heterocyclized compounds can be accommodated in the binding site, but conformational analysis showed that **11** can only bind in a higher-energy conformation. The amine orientation in **9**−**11** is shifted significantly to the side of D3.32 as compared to the reference (**5**), however the docking poses display near optimal interactions. This is because the interacting atoms both have flexible 1-carbon linkers and due to the shift in the scaffold placement. The 2-oxygen lone pairs in **8**−**11** have opposite directions to that of earlier constrained ligands.[15]–[17] The docked poses of **8**−**11** still display hydrogen-bonding to S3.36, but this requires a very specific positioning of the 2-oxygen with little flexibility and a slight of the scaffolds. The constraints in **9**−**11** resulted in docking poses with the 4-bromine in closer vicinity to 5.46, which is polar only in human 5-HT_2A_. Future medicinal chemistry programs should evaluate whether polar 4-substituted analogs can invert the target preferences.

## Methods

### Affinity measurements

K_i_ determinations were generously provided by the National Institute of Mental Health's Psychoactive Drug Screening Program, Contract # HHSN-271-2008-00025-C (NIMH PDSP). The NIMH PDSP is Directed by Bryan L. Roth MD, PhD at the University of North Carolina at Chapel Hill and Project Officer Jamie Driscol at NIMH, Bethesda MD, USA.

### Ligand docking into the 5-HT_2B_ crystal structure

The 5-HT_2B_ receptor crystal structure[25] in complex with the partial agonist ergotamine (4IB4) was downloaded from the protein data bank[27] and prepared with the Maestro protein preparation workflow[28]. A map of the interaction features and areas of the binding site was generated by SiteMap.[29] Accordingly, as a first step to adapt the binding pocket to phenylethylamine ligands, the hydroxyl hydrogen atoms of S3.36^139^ and S5.43^222^ were rotated towards the center of the binding site to constitute hydrogen bond donors. In opposite, the hydroxyl hydrogen of S3.37^140^ rotated away to enlarge a hydrophobic portion of the binding site. The triple-ring core of 2C-TCB (**5**) was better accommodated by tilting F6.52^341^ slightly towards TMH5 to the same position as observed in the G protein-bound β_2_ adrenergic structure.[26] The binding sites of the 5-HT_2_ receptor subtypes deviate only in two residue positions: 5.46 (5-HT_2A_: S, 5-HT_2B_: A and 5-HT_2C_: A) and 5.39 (5-HT_2A_: A, 5-HT_2B_: M and 5-HT_2C_: A). Models of the 5-HT_2A_ and 5-HT_2C_ binding sites were simply derived by mutation of these two residues.

Glide was used to generate the receptor grid, in which the S3.36 hydroxyl was allowed to rotate.[30] Glide SP was used for the docking, including sampling of ligand ring conformations and increasing the maximum number of output poses from 1 to 10. In a first docking round, (R)-Bromo-DragonFLY (**4**), (R)-2C-TCB (**5**) and **8**−**11** were docked with no further adjustments. In a second subsequent docking, separate grids were prepared for the reference and 1,2-constrained ligands, after shifting of the S3.36 rotamer to provide optimal fit (see Results). Binding poses were evaluated for placement of all interacting moieties as well as strain on the amine linker. For the reference ligands the top scoring poses proved satisfactory, whereas for **8**−**11** the selected poses were among the first 2−5 presented.

### Conformational penalties for strained ligand poses

The lowest energy (global minimum) conformations of **8**−**11** were calculated using MacroModel conformational searches with exhaustive settings (maximum iterations: 5000 and convergence threshold 0.01) and applying the OPLS2005 force field (used in all calculations). The energies of the bound conformations (all poses with a docking score within one unit of the highest scoring) were also calculated with MacroModel (current energy) after a mild minimization that restricted the movement of the heavy atoms to 0.3 Å. Finally, the conformational energy penalty of binding was calculated as the energy difference between the bound and global minimum conformations.

## Supporting Information

Methods S1 Detailed synthetic routes of 9, 10 and 11.
(DOC)
