# Correction: Structure-Activity Relationships of Constrained Phenylethylamine Ligands for the Serotonin 5-HT_2_ Receptors

**DOI:** 10.1371/annotation/c0047d59-f3e8-4923-8883-dcc991c0e61e

**Published:** 2014-01-17

**Authors:** Vignir Isberg, James Paine, Sebastian Leth-Petersen, Jesper L. Kristensen, David E. Gloriam

In the last paragraph of the Results section under the heading 'Differences in the binding site may explain the lower affinity of 8−11 in 5-HT_2A_ and higher affinity of 10−11 in 5-HT_2B_', the second sentence is incorrect. The correct sentence is: This position is occupied by a methionine in 5-HT_2B_, but an valine in 5-HT_2A_ and 5-HT_2C_ (Fig. 7a-c).

In the second paragraph of the Methods section under the heading 'Ligand docking into the 5-HT_2B_ crystal structure', the second to last sentence is incorrect. The correct sentence is: The binding sites of the 5-HT_2_ receptor subtypes deviate only in two residue positions: 5.46 (5-HT_2A_: S, 5-HT_2B_: A and 5-HT_2C_: A) and 5.39 (5-HT_2A_: V, 5-HT_2B_: M and 5-HT_2C_: V).

In the final paragraph of the Methods section, under the heading "Conformational penalties for strained ligand poses", the following sentence should be added as the last sentence of that paragraph: Marvin was used for drawing 2D structures (Marvin 5.12.3, 2013, ChemAxon, www.chemaxon.com) and 3D structures were illustrated using Pymol (PyMOL Molecular Graphics System, Version 1.5.0.4 Schrödinger, LLC).

Figure 7 is incorrect. The correct version of Figure 7 can be viewed here: http://plosone.org/corrections/pone.00078515.g007.cn.tif

