# Supplementary material for: Structure-Activity Relationships of Constrained Phenylethylamine Ligands for the Serotonin 5-HT2 Receptors
Source: PLoS One. 2013 Nov 7;8(11):e78515. doi: 10.1371/journal.pone.0078515 (PMC3820707; doi:10.1371/journal.pone.0078515)
Supplement: Methods S1 — Detailed synthetic routes of 9, 10 and 11. (DOC) [file pone.0078515.s001.doc]

# METHODS S1

Structure-Activity Relationships of Constrained Phenylethylamine Ligands for the Serotonin 5-HT_2_ Receptors

Vignir Isberg, James Paine, Sebastian Leth-Petersen, Jesper L. Kristensen, David E. Gloriam

Department of Drug Design and Pharmacology, Faculty of Health and Medical Sciences, University of Copenhagen, Copenhagen, Denmark

**Synthesis of 9**

2-(2-Bromo-4-methoxybenzyl)oxirane (12)

Cs2CO3 (11.2 g, 34.5 mmol, 1.5 equiv.) and epichlorohydrin (5.4 mL, 69 mmol, 3 equiv.) were added to a solution of 2-bromo-4-methoxyphenol (4.67 g, 23 mmol) in MeCN (100 mL) and the mixture heated to reflux for 4 h. The solvent was removed under reduced pressure and the residue partitioned between water (250 mL) and CH2Cl2 (100 mL). The organic layer was separated and the aqueous phase extracted with CH2Cl2 (2 x 75 mL). The combined organic extracts were washed with brine (200 mL), dried over Na2SO4 and concentrated under reduced pressure. The residue was purified by bulb-to-bulb distillation (0.2 mbar, oven temp. 180 - 200 °C) to give epoxide 12 (4.89 g, 82%) as a colourless oil. 1H NMR (300 MHz; CDCl3; Me4Si) δ 7.11 (1H, d, J 2.9, ArCH), 6.90 (1H, d, J 8.9, ArCH), 6.80 (1H, dd, J 8.9, 2.9, ArCH), 4.24 (1H, dd, J 11.2, 3.0, one of OCH2), 4.00 (1H, dd, J 11.2, 5.3, one of OCH2), 3.77 (3H, s, OCH3), 3.39 (1H, app. ddt, J 5.3, 4.1, 3.0, epoxide CH), 2.91 (1H, dd, J 5.0, 4.1, one of epoxide CH2) and 2.83 (1H, dd, J 5.0, 3.0, one of epoxide CH2); 13C NMR (75 MHz; CDCl3; CDCl3) δ 154.4 (C), 149.0 (C), 118.6 (CH), 115.5 (CH), 113.6 (CH), 113.0 (C), 70.7 (CH2), 55.8 (CH3), 50.1 (CH) and 44.6 (CH2).

(5-Methoxy-2,3-dihydrobenzofuran-3-yl)methanol (13)

n-BuLi (17.3 mL, 1.1 M, 1.1 equiv.) was added to a cooled (‑78 °C) solution of 12 (5.57 g, 21.5 mmol) in THF (100 mL). The reaction was allowed to warm to room temp. and stirred for a further 30 min before quenching with saturated aqueous NH4Cl solution (100 mL). The organic layer was separated, the aqueous phase extracted with CH2Cl2 (2 x 50 mL) and the combined organic layers dried over Na2SO4. Concentration under reduced pressure gave a residue that was purified by chromatography on silica (10–50 % EtOAc in petroleum ether) to provide the title compound (2.36 g, 61 %) as a pale yellow oil. 1H NMR (300 MHz; CDCl3; Me4Si) δ 6.82–6.80 (1H, m, ArCH), 6.72–6.70 (2H, m, ArCH), 4.63 (1H, app. t, J 9.0, one of CH2OH), 4.46 (1H, dd, J 9.0, 5.4, one of CH2OH), 3.82–3.73 (5H, m, OCH2 and OCH3), 3.60 (1H, app. dq, J 9.0, 6.0, CH) and 1.82 (1H, br. s, OH); 13C NMR (75 MHz; CDCl3; CDCl3) δ 154.4 (C), 153.9 (C), 128.0 (C), 113.5 (CH), 110.9 (CH), 109.5 (CH), 74.2 (CH2), 64.8 (CH2), 56.0 (CH3) and 45.0 (CH).

2-((5-Methoxy-2,3-dihydrobenzofuran-3-yl)methyl)isoindoline-1,3-dione (14)

Phthalimide (2.12 g, 14.4 mmol, 1.1 equiv.) and PPh3 (3.78 g, 14.4 mmol, 1.1 equiv.) were added to a solution of alcohol 13 (2.36 g, 13.1 mmol) in CH2Cl2 (50 mL). DEAD (2.27 mL, 14.4 mmol, 1.1 equiv.) was added dropwise over 10 min and the reaction was stirred for a further 1 h. The solvent was removed under reduced pressure and the residue purified by chromatography on silica (15–20 % EtOAc in petroleum ether) to give the title compound (3.75 g, 92%) as a colourless solid; mp 127–128 °C; 1H NMR (300 MHz; CDCl3; Me4Si) δ 7.89–7.85 (2 H, m, ArCH), 7.78–7.72 (2H, m, ArCH), 6.82–6.80 (1H, m, ArCH), 6.75–6.67 (2H, m, ArCH), 4.56–4.45 (2H, m, OCH2), 3.97–3.81 (3H, m, CH and CH2N) and 3.73 (3H, s, OMe); 13C NMR (75 MHz; CDCl3; CDCl3) δ 168.2 (C), 154.0 (C), 154.0 (C), 134.1 (CH), 131.7 (C), 127.9 (C), 123.4 (CH), 114.3 (CH), 110.6 (CH), 109.8 (CH), 75.2 (CH2), 56.0 (CH3), 42.4 (CH) and 41.4 (CH2).

(5-Methoxy-2,3-dihydrobenzofuran-3-yl)methanamine (15)

Hydrazine hydrate (1.89 mL, 61 mmol, 5 equiv.) was added to a hot solution of 14 (3.75 g, 12.1 mmol) in EtOH (100 mL). The solution was heated to reflux for 2 h, cooled to room temperature and diluted with Et2O (100 mL). The solids were removed by filtration, washed with Et2O, and the filtrate concentrated under reduced pressure. The solid residue was resuspended in Et2O (50 mL), the solids removed by filtration, and the filtrate concentrated under reduced pressure. The residue was purified by bulb-to-bulb distillation (0.3 mbar, oven temp. 140–160 °C) to give the title compound (1.44 g, 66%) as a colourless oil.

This was dissolved in EtOH (10 mL), acidified with concentrated aqueous HCl and diluted with Et2O (150 mL). The resulting crystals were removed by filtration, washed with Et2O and dried to provide the hydrochloride salt (1.64 g) as a colourless solid; mp 176–177 °C; 1H NMR (300 MHz; CD3OD; Me4Si) 6.95–6.91 (1H, m, ArCH), 6.76–6.64 (2H, m, ArCH), 4.60 (1H, dd, J 9.5, 6.8, one of OCH2), 4.45 (1H, dd, J 9.5, 4.9, one of OCH2), 3.84 - 3.70 (1H, m, CH), 3.73 (3H, s, OCH3), 3.28 (1H, dd, J 12.7, 4.5, one of CH2N and 3.10 (1H, dd, J 12.7, 9.0, one of CH2N); 13C NMR (75 MHz; CD3OD; CD3OD) δ 155.7 (C), 155.1 (C), 128.0 (C), 115.7 (CH), 111.6 (CH), 110.8 (CH), 75.5 (CH2), 56.4 (CH3), 43.8 (CH2) and 42.2 (CH).

(6-Bromo-5-methoxy-2,3-dihydrobenzofuran-3-yl)methanamine (9)

Br2 (1.6 mL, 1M in AcOH, 1.2 equiv.) was added dropwise to a cooled (5 °C) solution of the hydrochloride salt of 15 (292 mg, 1.35 mmol) in AcOH (20 mL). The mixture was allowed to warm slowly to room temp. and stirred for a further 18 h before pouring into water (100 mL). The solution was made strongly basic by the addition of 15 % aqueous NaOH and extracted with CH2Cl2 (3 x 50 mL). The combined organic extracts were dried over Na2SO4 and concentrated under reduced pressure. The residue was dissolved in EtOH (10 mL) and acidified by the addition of a 1 M solution of HCl in EtOH (2 mL). The crude product was precipitated by the addition of Et2O (100 mL), removed by filtration and recrystallized from EtOH/t-BuOMe to afford the hydrochloride salt of amine 9 (182 mg, 46%) as a colourless solid; mp 232–234 °C (dec.); (Found MH+, 260.0103. C10H1381BrNO2 requires M, 260.0104); 1H NMR (300 MHz; CD3OD; Me4Si) δ 7.07 (1H, s, ArCH), 6.99 (1H, s, ArCH), 4.63 (1H, dd, J 9.6, 8.7, one of OCH2), 4.45 (1H, dd, J 9.6, 4.9, one of OCH2), 3.83 (3H, s, OCH3), 3.80–3.70 (1H, m, CH), 3.29 (1H, dd, J 13.0, 4.7, one of CH2N) and 3.13 (1H, dd, J 13.0, 8.5, one of CH2N); 13C NMR (75 MHz; CD3OD; CD3OD) δ 155.5 (C), 152.0 (C), 127.4 (C), 115.2 (CH), 112.7 (C), 110.7 (CH), 76.1 (CH2), 57.6 (CH3), 43.6 (CH2) and 42.1 (CH).

**Synthesis of 10**

6-Methoxychroman-4-ol (17)

NaBH4 (1.2 g, 31.5 mmol, 1 equiv.) was added to a solution of 6-methoxychroman-4-one 16* (5.6 g, 31.5 mmol) in EtOH (50 mL). After stirring for 2 h the solvent was removed under reduced pressure and the residue partitioned between 1 M aqueous HCl (100 mL) and CH2Cl2 (50 mL). The organic layer was separated and the aqueous phase extracted with CH2Cl2 (2 x 25 mL). The combined organic phases were washed with brine, dried over Na2SO4 and concentrated under reduced pressure. The title compound was obtained as a pale yellow oil (5.03 g, 89%) and was used without further purification. 1H NMR (300 MHz; CDCl3; Me4Si) 6.80–6.78 (1H, m, ArCH), 6.74–6.71 (2H, m, ArCH), 4.64 (1H, t, J 4.0, CHOH), 4.17–4.12 (2H, m, OCH2), 3.70 (3H, s, OCH3), 2.72 (1H, br. s, OH), 2.11–1.99 (1H, m, CH2) and 1.97–1.88 (1H, m, CH2); 13C NMR (75 MHz; CDCl3; CDCl3) δ 153.3 (C), 148.4 (C), 124.6 (C), 117.7 (CH), 116.2 (CH), 113.5 (CH), 63.5 (CH), 62.1 (CH2), 55.8 (CH3) and 31.2 (CH2). *Selander H. and Nilsson J. L. G., Acta Chem. Scand., 1972, 26, 2433. (Tritium exchange in specifically labelled hydroquinone, dihydrobenzofuranol and chromanol derivatives).

6-Methoxychroman-4-carbonitrile (18)

Me3SiCN (4.98 mL, 40 mmol, 3 equiv.) was added to a solution of alcohol 17 (2.39 g, 13.3 mmol) in CH2Cl2 (125 mL). The solution was cooled to -50 °C and BF3.OEt2 (5.75 mL, 46.6 mmol, 3.5 equiv.) added dropwise. After stirring for 1 h the reaction was allowed to warm slowly to room temp. and quenched by the addition of saturated aqueous NaHCO3 (150 mL). The organic layer was separated and the aqueous phase extracted with CH2Cl2 (2 x 50 mL). The combined organic extracts were washed with brine (150 mL), dried over Na2SO4 and concentrated under reduced pressure. The residue was purified by chromatography on silica (5–10 % EtOAc in petroleum ether) to give nitrile 18 (2.44 g, 97%) as a yellow oil. 1H NMR (300 MHz; CDCl3; Me4Si) δ 6.78–6.76 (3H, m, ArCH), 4.26 (1H, ddd, J 10.9, 6.6, 4.3, one of OCH2), 4.19–4.11 (1H, m, one of OCH2), 3.98 (1H, t, J 6.1, CHCN), 3.75 (3H, s, OCH3) and 2.32–2.25 (2H, m, CH2); 13C NMR (75 MHz; CDCl3; CDCl3) δ 153.7 (C), 147.9 (C), 120.5 (C), 118.5 (CH), 116.6 (CH), 115.3 (C), 113.1 (CH), 63.6 (CH2), 55.9 (CH3), 27.2 (CH) and 26.3 (CH2).

(6-Methoxychroman-4-yl)methanamine (10)

DIBALH (7.5 mL, 42 mmol, 5 equiv.) was added to a solution of nitrile 18 (1.59 g, 8.4 mmol) in THF (50 mL). The reaction was heated to reflux for 2 h then quenched with sufficient 15 % aqueous NaOH to precipitate the aluminium salts as a white powder. This suspension was dried with Na2SO4, filtered and the filtrate concentrated under reduced pressure. The residual oil was purified by bulb-to-bulb distillation (0.5 mbar, oven temp. 180 °C) to give the title compound (1.32 g, 81 %) as colourless oil.

This was dissolved in EtOH (10 mL) and acidified with concentrated aqueous HCl. The solution was diluted with Et2O (100 mL) and the resulting crystals removed by filtration, washed with Et2O and dried to give 10.HCl (1.43 g) as a colourless solid; mp 178–180 °C; 1H NMR (300 MHz; CD3OD; Me4Si) δ 6.79–6.77 (1H, m, ArCH), 6.74–6.66 (2H, m, ArCH), 4.14–4.09 (2H, m, OCH2), 3.72 (3H, s, OCH3), 3.36 (1H, dd, J 11.8, 3.3, one of CH2N), 3.25–3.15 (1H, m, CHCH2N), 3.12 (1H, dd, J 10.4, 11.8, one of CH2N), 2.18–2.05 (1H, m, one of OCH2CH2) and 2.03–1.92 (1H, m, one of OCH2CH2); 13C NMR (75 MHz; CD3OD; CD3OD) δ 154.8 (C), 150.0 (C), 122.4 (C), 118.8 (CH), 115.9 (CH), 114.3 (CH), 63.4 (CH2), 56.2 (CH3), 45.1 (CH2), 33.7 (CH) and 25.7 (CH2).

**Synthesis of 11**

4-(3-Bromo-4-methoxyphenoxy)butanoic acid (20)

Ethyl 3-bromobutyrate (3.9 mL, 27.3 mmol, 1.1 equiv.) was added to a suspension of 3-bromo-4-methoxyphenol (19)** (5.03 g, 24.8 mmol) and Cs2CO3 (8.9 g, 27.3 mmol, 1.1 equiv.) in MeCN (75 mL). The reaction was heated to reflux for 2 h and the solvent removed under reduced pressure. The residue was partitioned between water (250 mL) and CH2Cl2 (100 mL) and the organic layer separated. The aqueous phase was extracted with CH2Cl2 (2 x 50 mL) and the combined organic phases washed with brine (100 mL). The solvent was removed under reduced pressure and the crude product purified by bulb-to-bulb distillation (0.5 mbar, oven temp. 210 - 220 °C) to give the ester (7.18 g, 91 %) as a colourless oil.

The ester (7.0 g, 22 mmol) was dissolved in MeOH (25 mL) and NaOH (1.06 g, 26.4 mmol, 1.2 equiv.) added. The solution was heated to 60 °C for 15 min then poured into 150 mL 1 M HCl. The precipitate was removed by filtration and dried to provide the title compound (5.91 g, 93%) as a colourless solid; mp 98-100 °C; 1H NMR (300 MHz; CDCl3; Me4Si) δ 7.09 (1H, dd, J 2.3, 0.8, ArCH), 6.83–6.75 (2H, m, ArCH), 3.94 (2H, t, J 6.1, OCH2), 3.82 (3H, s, OCH3), 2.56 (2H, t, J 7.1, CH2CO2H) and 2.08 (2H, tt, J 7.1, 6.1, OCH2CH2); 13C NMR (75 MHz; CDCl3; CDCl3) δ 179.6 (C), 153.0 (C), 150.3 (C), 119.7 (CH), 114.3 (CH), 112.9 (CH), 111.9 (C), 67.4 (CH2), 57.0 (CH3), 30.7 (CH2) and 24.5 (CH2). **Wubbels, Gene G.; Brown, Toby R.; Babcock, Travis A.; Johnson, Kandra M.
Journal of Organic Chemistry,   2008 ,  vol. 73,  # 5   p. 1925 – 1934.

8-Bromo-7-methoxy-3,4-dihydrobenzo[b]oxepin-5(2H)-one (21)

4-(3-Bromo-4-methoxyphenoxy)butanoic acid (20) (2.78 g, 9.6 mmol) was finely ground and added to polyphosphoric acid (50 mL). The mixture was heated to 90 °C for 1 h, stirring occasionally with a glass rod, before pouring into ice-water (200 mL). This was stirred until all the oily material dissolved and the insoluble product removed by filtration. The crude product was crystallised from aqueous EtOH to give the title compound (1.88 g, 72%) as a yellow solid; mp 104-106 °C; 1H NMR (300 MHz; CDCl3; Me4Si) δ 7.32 (1H, s, ArCH), 7.24 (1H, s, ArCH), 4.18 (2H, t, J 6.7, OCH2), 3.89 (3H, s, OCH3), 2.91–2.85 (2H, m, CH2C=O) and 2.22–2.11 (2H, m, OCH2CH2); 13C NMR (75 MHz; CDCl3; CDCl3) δ 199.6 (C), 155.5 (C), 151.8 (C), 128.6 (C), 126.3 (CH), 117.8 (C), 110.4 (CH), 73.1 (CH2), 56.8 (CH3), 40.5 (CH2) and 25.8 (CH2).

8-Bromo-7-methoxy-2,3,4,5-tetrahydrobenzo[b]oxepin-5-ol (22)

NaBH4 (231 mg, 6 mmol, 1 equiv.) was added to a suspension of ketone 21 (1.65 g, 6 mmol) in EtOH (20 mL). This was stirred for 1 h, during which time the ketone went into solution, then the solvent was removed under reduced pressure. The residue was partitioned between 1 M aqueous HCl (100 mL) and CH2Cl2 (75 mL). The aqueous phase was extracted with CH2Cl2 (2 x 50 mL), the combined organic layers washed with brine and dried over Na2SO4. The solvent was removed under reduced pressure and the crude product crystallised from EtOAc/petroleum ether to yield the alcohol (1.49 g, 91%) as a colourless solid; mp 90-93 °C; 1H NMR (300 MHz; CDCl3; Me4Si) δ 7.18 (1H, s, ArCH), 6.97 (1H, s, ArCH), 4.87–4.79 (1H, m, CHOH), 4.13–4.04 (1H, m, one of OCH2), 3.85 (3H, s, OCH3), 3.80–3.70 (1H, m, one of OCH2), 2.47 (1H, d, J 6.0, OH) and 2.11–1.77 (4H, m, OCH2CH2CH2 and OCH2CH2CH2); 13C NMR (75 MHz; CDCl3; CDCl3) δ 152.3 (C), 151.6 (C), 137.6 (C), 126.4 (CH), 110.0 (CH), 109.3 (C), 73.6 (CH2), 72.2 (CH), 56.8 (CH3), 34.7 (CH2) and 27.7 (CH2).

8-Bromo-7-methoxy-2,3,4,5-tetrahydrobenzo[b]oxepine-5-carbonitrile (23)

BF3.OEt2 (2.3 mL, 18.9 mmol, 3.5 equiv.) was added dropwise to a cooled (-78 °C) solution of alcohol 22 (1.48 g, 5.4 mmol) and Me3SiCN (2.0 mL, 16.2 mmol, 3 equiv.) in CH2Cl2 (100 mL). The reaction was allowed to warm slowly to room temp. and stirred for a further 2 h before quenching with saturated aqueous NaHCO3 (100 mL). The organic layer was separated and the aqueous phase extracted with CH2Cl2 (2 x 50 mL). The combined organic layers were washed with brine, dried over Na2SO4 and concentrated under reduced pressure. The residue was purified by chromatography on silica (10 % EtOAc in petroleum ether) to give the title compound (810 mg, 53%) as a pale yellow oil. 1H NMR (300 MHz; CDCl3; Me4Si) δ 7.25 (1H, s, ArCH), 6.91 (1H, s, ArCH), 4.10–3.97 (2H, m, OCH2), 3.92–3.83 (1H, m, CHCN), 3.87 (3H, s, OCH3) and 2.25–1.92 (4H, m, OCH2CH2CH2 and OCH2CH2CH2); 13C NMR (75 MHz; CDCl3; CDCl3) δ 152.9 (C), 152.2 (C), 128.8 (C), 127.1 (CH), 119.4 (C), 111.4 (CH), 110.9 (C), 73.1 (CH2), 56.7 (CH3), 35.1 (CH), 30.0 (CH2) and 29.8 (CH2).

(8-Bromo-7-methoxy-2,3,4,5-tetrahydrobenzo[b]oxepin-5-yl)methanamine (11)

DIBALH (2.4 mL, 13.7 mmol, 5 equiv.) was added to a solution of nitrile 23 (772 mg, 2.7 mmol) in THF (50 mL). The reaction was heated to reflux for 2 h then quenched with sufficient 15 % aqueous NaOH to precipitate the aluminium salts as a white powder. This suspension was dried with Na2SO4, filtered and the filtrate concentrated under reduced pressure. The residue was dissolved in EtOH (5 mL) and a 1 M solution of HCl in EtOH (3 mL) added. The solution was diluted with Et2O (100 mL) and the precipitate removed by filtration, washed with Et2O and dried to afford the hydrochloride salt of the title compound (584 mg, 66 %) as a colourless solid; mp 224-226 °C; (Found MH+, 288.0388. C12H1781BrNO2 requires M, 288.0417); 1H NMR (300 MHz; CD3OD; Me4Si) δ 7.17 (1H, s, ArCH), 6.91 (1H, s, ArCH), 4.32 (1H, dtd, J 11.9, 3.4, 1.1, one of OCH2), 3.86 (3H, s, OCH3), 3.54 (1H, dt, J 11.9, 1.8, one of OCH2), 3.42 (1H, dd, J 12.2, 9.4, one of CH2N), 3.33–3.16 (2H, m, CH and one of CH2N), 2.25–2.03 (2H, m, one of OCH2CH2CH2 and one of OCH2CH2CH2) and 1.91–1.74 (2H, m, one of OCH2CH2CH2 and one of OCH2CH2CH2); 13C NMR (75 MHz; CD3OD; CD3OD) δ 154.9 (C), 153.7 (C), 134.7 (C), 128.0 (CH), 115.0 (CH), 110.9 (C), 75.0 (CH2), 57.2 (CH3), 44.2 (CH), 42.3 (CH2), 29.3 (CH2) and 28.0 (CH2).
